# “Organismic” positions in early German-speaking ecology and its (almost) forgotten dissidents

**DOI:** 10.1007/s40656-020-00328-9

**Published:** 2020-09-30

**Authors:** Kurt Jax

**Affiliations:** 1 Department of Conservation Biology, Helmholtz-Centre for Environmental Research - UFZ, Permoserstraße 15, 04318 Leipzig, Germany; 2 Department Ecology and Ecosystem Management, Technical University of Munich, Emil-Ramann-Str 6, 85354 Freising, Germany

**Keywords:** Biocoenosis, Community concept, Holism, Ecological units

## Abstract

In early German ecology, the key concept used to refer to a synecological unit was *Biozönose* (biocoenosis). Taken together with the concept of the *Biotop* (biotope), it was also understood as an integrated higher-order unit of life, sometimes called a “*Holozön*” (holocoen). These units were often perceived as having properties similar to those of individual organisms, and they informed the mainstream of German ecology until at least the late 1960s. Here I ask how “organismic” these concepts really were and what conceptual problems they entailed. To do so, I focus on some almost forgotten dissident positions, especially those of (German-born) Friedrich Simon Bodenheimer and Fritz Peus, which I contrast with the mainstream German ecology of the time. In a radical paper published in 1954 that postulated the “dissolution of the concepts of biocoenosis and biotope”, Peus in particular elicited a forceful response from many prominent German ecologists. An analysis of the ensuing debate, including especially a colloquium held in 1959 that was partly inspired by Peus’ paper, is helpful for sifting the various arguments proffered with respect to a quasi-organismic perception of the biocoenosis in German speaking ecology. Although German mainstream ecologists rejected the notion of the biocoenosis as a superorganism, ontological holism was quite common among them. Additionally, the mainstream concept of the biocoenosis was plagued by several methodological problems and much conceptual confusion, to which the “dissidents” rightly pointed. Some of these problems are still pertinent today, e.g. in connection with more modern concepts such as “ecosystem”.

## Introduction

The early decades of ecology in the German-speaking world^1^ were dedicated not only to gathering extensive empirical knowledge but also to forming concepts and theories in order to construct an interpretative framework for ecological knowledge. Among the various concepts created to describe (syn)ecological units, i.e. units formed by individuals from different species in a given location,^2^ that of the *Biozönose* (biocoenosis) was a key one. It had been developed by Karl August Möbius (1825–1908) in the course of his study on oyster beds and on whether it was possible to grow oysters commercially in the German Wadden Sea (Möbius 1877; see Potthast 2020). Referring to his studies of the oyster beds Möbius defined the biocoenosis as follows:

Science possesses, as yet, no word by which such a community of living beings may be designated; no word for a community where the sum of species and individuals, being mutually limited [1877: interdependent (*sich bedingen*)] and selected under the average external conditions of life, have, by means of transmission, continued in possession of a certain definite territory [1877: perpetuate themselves in a particular area (*sich in einem abgemessenen Gebiete dauernd erhalten*)]. I propose the word Biocoenosis [1877: Biocoenosis or living community (*Biozönose oder Lebensgemeinde*)] for such a community. (Möbius 1883, p. 723/1877, p. 75f)^3^

Some years later Friedrich Dahl (1904, 1908) coined the term *Biotop* (biotope) to refer to the topographic, abiotic counterpart to the biocoenosis. The biotope, as it was and is perceived in German-speaking ecology, can be understood as the habitat not of a single species but of the whole biocoenosis (see Jax 2002, chapter 3.3 for details). Taken together, biotope and biocoenosis were also perceived as an integrated higher-order unit of life, sometimes called “*Holozön*” (holocoen; Friederichs 1927, 1937; see Jax 1998; Potthast 2020). Biocoenosis and units of still higher order were often perceived as having properties similar to those of an individual organism, such as self-regulation, the mutual dependency of its parts, or “harmony” (see below) and formed the mainstream of German ecology until at least the late 1960s.^4^

Both German and English-speaking authors often saw the word “biocoenosis” as equivalent to and/or translated it as (ecological) “community”. This simple translation, however, only holds for a restricted set of understandings of the much broader concept of community. The concept of community is used for a variety of different notions and concepts (see Jax et al. 1998; Jax 2006), ranging from Robert MacArthur’s famous definition of the community as “any set of organisms currently living near each other and about which it is interesting to talk” (MacArthur 1971, p. 190) to highly specialised definitions where the properties necessary to call an assemblage of species a “community” came close to those postulated for the biocoenosis (e.g. Clements 1916; Allee et al. 1949).^5^ Thus a simple translation of “biocoenosis” as “community” is not justified.

In any case, the biocoenosis has often been perceived (and also criticised—see below) as a holistic or, even more, an organismic concept. In this paper I will ask how holistic and “organismic” this concept really was (as perceived by its proponents) and which conceptual problems it entailed. In order to do so, I will focus on some almost forgotten dissident positions, especially those of (German-born) Friedrich Simon Bodenheimer (1897–1959) and Fritz Peus (1904–1978), which I will contrast with the mainstream of German ecology. In a radical paper published in 1954 that postulated nothing less than the “dissolution of the concepts of biocoenosis and biotope”, Peus in particular elicited a forceful response from many prominent German ecologists. By juxtaposing the positions of Peus and Bodenheimer with those of the German mainstream, I seek to give a voice to those who were in a minority position against widespread “agreement” on the nature of the biocoenosis in German ecology. More than this, though, I use Peus’ and Bodenheimer’s arguments—and especially the responses of mainstream authors to their critique—to sharpen our apprehension of the conceptual rigour and robustness of the “organicist” approaches of German mainstream ecologists. This helps to elucidate the well-known statements found in papers and textbooks written by prominent ecologists such as Thienemann and Friederichs. In defending their positions against powerful critique, the mainstream ecologists had to clarify and justify their sometimes rather vague postulates. An analysis of the ensuing discussion thus is helpful for sifting the various arguments proffered with respect to a quasi-organismic perception of the biocoenosis in German-speaking ecology.

## The context: organicism and holism in early 20th century ecology^6^

In spite of some slight variations in the definition and understanding of the biocoenosis concept, and debates about the classification and subdivision of biocoenoses, the Hungarian ecologist János Balogh was still able to state in his 1958 textbook:

In the eight decades which have passed since the work of Möbius, Möbius’ concept of the biocoenosis has in essence been left unchanged, even though knowledge about the biocoenoses has grown considerably during that time, and even though wide-ranging developments in theory—sometimes perhaps of a somewhat speculative kind—have unfolded (p. 17; translations from German here and in the following by KJ).^7^

What Balogh hints at in the last part of his sentence is the fact that German ecologists in the first half of the 20th century were increasingly preoccupied with the philosophical underpinnings of the biocoenosis concept and that of other ecological units. Considerations about the “nature” of the biocoenosis were situated within the context of a broader debate about the unity of nature and the unity of science. In biology, the main opposition was one between mechanism at the one extreme and vitalism at the other. Mechanism, understood as the attempt to reduce all phenomena of life to the physical and chemical interactions of their components and thus to provide a sufficient explanation, a “machine theory” of life, was perceived as being just as inadequate as (neo)vitalism. Vitalism postulated an immaterial life force—or entelechy, in the terminology of its main proponent Hans Driesch—to explain the phenomenon of life. Such a life force, it was claimed, was irreducible to physics and chemistry and was invoked to explain finality and purposiveness in the realm of the organic.

Organicism was seen by its adherents as a third way between the two extremes, an alternative understanding of life that claimed to form a synthesis out of mechanism and vitalism. The organic, holistic worldview emphasized the “organic wholeness” of the world (Meyer-Abich 1941) and was expected to put (back) together what had been broken apart by the analytical, dissecting sciences such as physics, chemistry and physiology. The approaches taken by the different proponents of organicism are very heterogeneous.^8^ However, common to all these theories is an emphasis on “organisation”, that is, the internal relations between the component parts of wholes, which are seen as fundamental to understanding the wholes. Organisation was perceived as being neither just a sum of components nor a postulated mysterious immaterial life force. The way in which “wholes” are organised was regarded as similar to the organisation of the individual organism. Such a view was also applied to the inorganic realm and to psychological and social phenomena. Some of the more prominent (although theoretically diverse) proponents of organicism within biology were, for example, J. S. Haldane, Joseph Needham, Ludwig von Bertalanffy, Adolf Meyer-Abich, and Karl Friederichs.^9^

Some of the philosophical foundations of these ideas have a long history. Especially in Germany they were influenced strongly by the Romantic natural philosophy (*Naturphilosophie*) of the nineteenth century. The most prominent and influential German ecologists of the early 20th century who subscribed to such ideas were August Thienemann^10^ (1882–1960) and Karl Friederichs^11^ (1878–1969).^12^ Both were highly inclined toward philosophical thinking. Thienemann’s habilitation thesis, for example, was entitled: “*Die Stufenfolge der Dinge, der Versuch eines natürlichen Systems der Naturkörper aus dem achtzehnten Jahrhundert*” (“The graduation of things: An attempt at a natural system of natural bodies from the eighteenth century”) (Thienemann 1909), while Friederichs, even more than Thienemann, pursued a philosophical quest to understand nature and the postulated unity of nature (e.g. Friederichs 1927, 1937, 1955).

For “the” German ecologists, perceiving the unity of nature (that is, grasping its “wholeness” conceptually) was both the starting point and ultimate aim of ecological research. Ecology was considered by its protagonists to belong to a type of science that was different from “traditional” science. The analytic/reductionistic approach of “traditional” science was contrasted to the “viewing” or “observing” (“*schauende*”) approach of a synthetic approach oriented toward German *Naturphilosophie*. Friederichs and Thienemann referred to Galileo and Newton on the one hand and to Goethe on the other to illustrate these different approaches toward nature. The former two represented the approach of “analytical cognition”, looking for laws and causal relations; the latter was seen as the key proponent of a “viewing” examination, the aim of which is to perceive “*Gestalten*” (in the sense of Gestalt psychology^13^). To Friederichs, the wholeness of nature and of its purposeful order was simply evident (“…and we would have to be blind not to realize that all is arranged in mutual order to each other and in mutual dependence”; Friederichs 1927, p. 156).^14^ This view had important consequences for his approach to the scientific understanding of nature. For him, nature as a whole with its “naturally delimited parts” could not be conceived of as a sum of its parts, as in the mechanistic worldview, but only as wholes or “*Gestalten*”. He saw one such wholeness in the biocoenosis, which he understood as a “unit of life” (*Lebenseinheit*)—in the sense of earlier definitions of the biocoenosis (e.g. by Möbius)—or as a “biological system which actively persists by self-preservation” (“*biologisches System, das sich durch Selbsterhaltung bei Bestand erhält*”; Friederichs 1927, p. 155). He wrote: “In the same way that the world is a dynamic system, which actively persists in a delicate state of equilibrium by means of self-regulation, this also holds for naturally delimited parts of the biosphere (e.g. pond, fen, beach)…. [A]nd all this life together constitutes a set of textured relations: the biocoenosis.” (Friederichs 1937, p. 18)^15^ “… und all dieses Leben zusammen bildet ein Beziehungsgefüge: die Lebensgemeinschaft.” Likewise, Thienemann subscribed to a holistic and “viewing” approach to nature, stating: “The presentation of the ‘Lebenseinheiten’ [‘units of life’] as I have done here—their ‘development’ from the single organism via the biocoenosis to the holocoen—this holistic view of nature is without doubt a ‘viewing’ perception of nature of a morphological kind” (Thienemann 1954, p. 317).^16^ In terms of the character of these units of life, he repeatedly mentions that one could perceive of biocoenoses—and indeed whole lakes (a biocoenosis together with its biotope)—”*gleichsam*” (“so to speak”) as “ higher-order organisms” (“*Organismen höherer Ordnung*” e.g. Thienemann and Kieffer 1916; Thienemann 1926, 1954).

## Bodenheimer, Peus and the critique of the biocoenosis concept

Such “organismic” perceptions of the biocoenosis constituted the mainstream of German ecology, and few German ecologists expressed any fundamental concerns about this approach. Of the few critics of the biocoenosis concept during the early and mid-20th century, Bodenheimer and Peus can be considered the most radical ones.^17^

Friedrich Simon (Frederick Shimon) Bodenheimer (1897–1959) was born in Cologne. He studied at the Universities of Munich and Bonn, moving into applied entomology and studying with, among others, zoologist and animal geographer Richard Hesse. He graduated—as a student of Hesse—in Bonn in 1919. Highly sympathetic to the Zionist cause,^18^ Bodenheimer emigrated to Palestine in 1922, where he held the first post in entomology at the Agricultural Experiment Station in Tel Aviv and, from 1928, at the University of Jerusalem. Later in Israel he became somewhat of a doyen in animal ecology and entomology for his country (Harpaz 1984). However, Bodenheimer was not only an ardent empirical researcher, he also always had a strong interest in the history and philosophy of biology and ecology.

As an animal ecologist concerned with pest management, Bodenheimer dealt with, among other things, the question of how animal populations are regulated. Early on (see Bodenheimer 1928)^19^ he became an eager proponent of the density-independent regulation of populations, emphasizing abiotic factors, especially climate, as the main factors responsible for keeping animal numbers within certain limits, as opposed to “density-dependent” biotic factors such as competition, predation or parasitism (see e.g. Sinclair 1989; Murray 1999 for the longstanding debate on population regulation). This is in sharp contrast to the idea of the biocoenosis as a self-regulating, harmonious unit in which populations were kept in balance by each other or even by an overarching “ecological unifying factor” (“*ökologischer Einheitsfaktor*”) (e.g. Friederichs 1927, 1930). The background for this was Bodenheimer’s self-declared adherence to “mechanistic principles” (“*mechanistische Prinzipien*”) (Bodenheimer 1928, p. 736) for explaining ecological patterns and processes. At the end of his early paper on the regulation of insect populations, for example, he stated:

On account of this basic position alone, the principles set out here will evoke lively opposition from the present-day biocoenologists, who already state that the genius loci or the ecological unifying factor is more than the sum of all single causal factors of an ecological unit (p. 736).^20^

Bodenheimer thus rejected outright the popular ideas about the biocoenosis (outlined above) as being too speculative and not grounded in sound empirical research but often more in “romantic” philosophy (Bodenheimer 1958, p. 156, FN), which he—with some justification (see above)—saw as the background of many German ecologists. Bodenheimer did not share the assumption of the latter that the existence of the biocoenosis as an integrated whole was a proper starting point for ecological research. He accuses the German ecologists of relying too much on intuition and (organismic) analogies. Bodenheimer characterised his epistemology—to which he devotes some space in his writing^21^—and methodology as a mechanistic and empirical one. For him, the proper approach to analysing ecological communities was one based on strong empirical research (experiment and observation), with some degree of intuition serving only as an initial guide to such empirical research (e.g. Bodenheimer 1938, p. 142). While Bodenheimer still allowed for a heuristic role of the supraorganismic concept (e.g. Bodenheimer 1957, p. 87), the supraorganismic properties of communities postulated by the ecologists were only one possible result of research for him, not a proven fact to build on:

No partisan of the empirical school has to our knowledge ever denied the possibility of a supraorganismic structure of bio-communities. This school only has stressed that at the present state of our knowledge the factual basis of the analysis of the ‘web of nature’ is entirely inadequate for a general synthetic solution. (Bodenheimer 1957, p. 86)

He is especially sceptical about the “almost religious enthusiasm amongst ecologists” (ibid) with respect to the apparent harmony of living nature and the comparison made between the holistic perception of nature and listening to music (instead of hearing only the single notes):

German ecologists have compared the supraorganismic hypothesis of the bio-communities to listening to the ‘symphony of spheres’, a well-chosen simile stressing the intuitive character of this theory. It is obvious that no scientific method can arrive at a perception of this symphony of the spheres. It is in this sense, and in this sense only ‘that we would gladly accept the accusation of being a non-musical person, unable to perceive the music of nature’s composition.’^22^ (ibid)

Bodenheimer undertook an elaborate exploration of the nature of the biotic community and, specifically, the biocoenosis in his book “Problems of animal ecology” (1938), later extended and slightly revised as “Animal ecology to-day” (1958)^23^ and in a paper from 1957 entitled: “The concept of biotic organization in synecology”.^24^ Bodenheimer did not deny that ecological communities “exist”, but he considered them as “a chance one which is created by history and selection” (Bodenheimer 1958, p. 189). He further states:

There is no doubt that there exist animal communities of different orders, characterized by the abundance of some species, the dominants, and the presence of mostly only a few individuals of other species more or less restricted to one community only, the characteristic species. But each member is a more or less independent member of the community, existing in it by right of its own ecoworld (ibid).

He is thus closer to the concept of the statistical communities used by Petersen (1913) to describe the benthic communities of the Danish seabed, which he discusses at some length, than to the more ambitious concept of the biocoenosis and its organismic analogies.

Beyond the more epistemological objections described, Bodenheimer’s critique of the biocoenosis concept refers additionally to a number of other points.

It is highly likely that part of the reason for his rejection of the biocoenosis concept as an established fact is to be found in his insistence on population regulation as being density-independent, a position that was partly shaped by the experiences he had as an entomologist in the often very harsh environments of the eastern Mediterranean, especially Israel, where he worked. As a result, he regarded biotic interactions—the core of the biocoenosis idea—as being decidedly limited in their significance (see Harpaz 1984 p. 16f. also Bodenheimer 1959, p. 361^25^).

Another explicit argument to be found in Bodenheimer’s writings relates to Thienemann’s comparison of the nutrient cycles of lakes with the physiology or metabolism of an individual organism, thus creating the impression that a lake is a “higher-order organism” (Thienemann 1925). He argues:

This system [here: a lake] is therefore not to be compared with the metabolism of an organism which requires purposely and selectively the materials necessary for the maintenance of the organism from outside, and which has a complicated physiological system of internal dissimilation, transport and distribution of matter. One phase of the circulation interrupted, the organism breaks down. However, in the lake, life, perhaps in the form of another life community, would be maintained if no fish were present of [sic! intended: or] if bacterial composition were quite incomplete. (Bodenheimer 1957, p. 84f)

In addition, Bodenheimer argues (1957, p. 85), the constancy and continued existence of species assemblages cannot be taken as proof of the “supra-organismic organisation of the bio-community” because it can often also simply be explained by a constancy in environmental conditions.^26^

When Bodenheimer states, as quoted above, that “each member [of the animal community] is a more or less independent member of the community, existing in it by right of its own ecoworld”, his use of the neologism “ecoworld” is a reference to Jakob von Uexküll (see also Bodenheimer 1958, p. 164f, for an explicit reference) with his idea of the “*Umwelt*” as a *subjective* world proper to each species, or even each individual. This “*Umwelt*” is very different from the common notion of a general biophysical environment (which would be the literal translation of Uexküll’s more specific technical term “*Umwelt*”) and again puts the individual species or organism at the centre of Bodenheimer’s approach. It is Fritz Peus, however, who takes up some of Uexküll’s ideas and moves toward an even more radical critique of the biocoenosis concept.^27^

In contrast to Bodenheimer, Fritz Peus (1904–1978) was less known for any philosophical or historical reflections on ecology and its concepts—with one highly notable exception, which I will discuss now. Friedrich (Fritz) Peus was born in Siegen (Westfalia). He studied at the Universities of Münster and Rostock, becoming like Bodenheimer and their adversary Karl Friederichs, an applied zoologist and dealing with (agricultural) pests and their control.^28^ Later in his career he became the director of the Berlin Museum of Natural History and Professor for Zoology at Humboldt University in the eastern part of Berlin. Around the time the Berlin wall was built (August 13, 1961), he moved from this post, continuing as a professor and director of Applied Zoology at the Free University of Berlin in West Berlin (April 1962), a position he held until his retirement in 1969 (Schumann 1980).

In 1954 Peus published a paper entitled “*Auflösung der Begriffe ‘Biotop’ und ‘Biozönose’*”. (“Dissolution of the concepts ‘biotope’ and ‘biocoenosis’”).^29^ An analysis of this paper and especially of the (published) responses to it provide interesting insights that help us better understand the general ideas held by German ecologists about their central concept, the biocoenosis.

In his paper, Peus first describes his own ideas about the relations between the (individual) organism, specifically the animal, and its environment.^30^ He then goes on to criticize and “dismantle”, piece by piece, the concepts of the biotope and biocoenosis, the latter by reference to what he considers to be the main properties commonly ascribed to the biocoenosis.

Peus’ approach is based on an autecological perspective on ecology. For him, the starting point of a scientific view on animal “communities” must always be the *individual species* (or even individual organism) and its specific ecological environment (“*ökologische Umwelt*”^31^). Making use of Uexküll’s terminology^32^ as well as ideas about the ecological niche (with ideas similar to Hutchinson’s niche concept, which was, however, proposed only four years later, in 1957) Peus thus defined the (ecological) *Umwelt* as follows:

Unter der *ökologischen Umwelt* möchte ich ausschließlich die Gesamtheit derjenigen Faktoren verstehen, auf die eine Spezies, natürlich auf dem Wege über ihre Individuen, für ihre Existenz und für ihr Gedeihen angewiesen ist. Die Umwelt ist grundsätzlich unabhängig von einem bestimmten Raum.

By the *ecological Umwelt* I understand exclusively the totality of those factors on which a species, of course by way of its individuals, is dependent for its existence and its thriving. The *Umwelt* is fundamentally independent of a specific space (p. 274).

Each species thus has its unique *Umwelt, depending on its specific morphology and physiology;* there is no overarching general biophysical environment which is relevant to all organisms in the same way.^33^ Additionally, for Peus distinguishing between biotic and abiotic factors of the *Umwelt* makes no sense from the perspective of an animal: all these factors are of fundamentally equal relevance in their effects.

It is from this vantage point that Peus criticizes the concepts of the biotope and the biocoenosis. With respect to the biotope he argues that everything that is to be said about the possibility of the common occurrence of species in a given place can be said simply by referring to the nature of their *Umwelten,* and that the concept of the biotope is therefore unnecessary and is not based on scientific evidence (p. 289f).

Der überkommene Begriff des Biotops ist also weiter nichts als eine menschliche Bezeichnung des Rahmens, der Umhüllung, der ‚Kulisse‘ für die Umwelten der Tiere (p. 289f).

The received concept of the biotope is thus nothing more than a human description of the frame, the envelopment, the ‘scenery’ for the *Umwelt*s of the animals.

In the third part of his text Peus criticizes and deconstructs the concept of the biocoenosis by first describing and then refuting what he sees as the major characteristics of the biocoenosis concept. He substantiates his claims about these main (putative) characteristics of the biocoenosis using quotes from Möbius, Reswoy (1924)^34^ and especially his contemporaries Thienemann and Friederichs, all of them highly influential ecologists.

What, then, were the essential characteristics of the biocoenosis concept which Peus extracted from the literature? He names four:

From the above quotes we can extract […] the following as essential characteristics of the biocoenosis concept:

**Closedness**: saturation—autarchy (self-reliance, independence);

**Community (for its members)**: mutual conditionality—fixed bonding with each other—dependence on each other—with each other, for each other, against each other—everything being connected with everything else;

**Equilibrium:** ability to self-regulate, to self-preserve in perpetuity, to keep itself in existence—relatively constant mutual proportions between the numbers of species and individuals, remaining in balance—balance between production, consumption and decomposition;

**Unity:** harmony—biological system—organisation (‘higher-order organism’; ‘higher-level organic individuality’)—wholeness—consequently: living beings as ‘members’ rather than parts of this unit (p. 295).^35^

Previous authors (e.g. Caspers 1950) had already pointed at the lack of *closedness* of the “type specimen” of the biocoenosis, the oyster bed. Because oysters feed on plankton and because their own planktonic larvae are transported over wider areas, it is almost impossible to determine the boundary of the oyster bed on the basis of its interactions; thus the oyster bed is not a spatially closed unit, and neither are other ecological communities.

Likewise, Peus does not see a “*community*”^36^ in terms of its members, in the sense of mutual dependence and a specific relation between organisms “with each other, for each other, against each other”. With the exception of symbioses, he sees all other relations between animals as unilateral in the sense of one or the other benefiting or being disadvantaged respectively. He concludes (going back to his own approach):

[…] daß *jedes Lebewesen* (als Spezies) *auf sich allein gestellt ist, für sich allein steht* und an einem Ort oder Zeit gedeiht oder kümmert *nach Maßgabe der Beschaffenheit* *seiner* *Umwelt*. In dieser Schau ist der Begriff der Gemeinschaft nicht am Platze (p. 297).

[…] that *each living being* (as a species) *is on its own, stands for itself* and flourishes or dies back at a place or time *depending on the conditions of* *its* *Umwelt*. In this view, the concept of community is out of place (p. 297).

*Equilibrium*, not least in the sense of a *self*-*regulation* of the biocoenosis as characterised by Möbius, is likewise regarded by Peus as non-existent, as he does not see constancy but rather change as being the rule in nature:

Da […] jede Spezies bzw. Population nach Maßgabe der (sich ändernden) Beschaffenheit ihrer Umwelt auf sich allein gestellt lebt, sehen wir keine Möglichkeit, von einer Selbstregulation seitens der Gesamtheit der anwesenden Organismen zu sprechen; es ist nichts da, was diese Funktion steuern sollte, worin eine Selbsterhaltung des Ganzen begründet liegen und worauf sich ein Bei-Bestand-Halten beziehen könnte (p. 297).

As […] each species or population lives completely on its own, depending on the (changing) conditions of its *Umwelt*, we see no possibility of speaking of self-regulation on the part of the totality of the organisms present; there is nothing there which could steer this function, nothing on which a self-preservation of the whole could be based, nothing to which a keeping-itself-in-existence could refer (p. 297).

Finally, Peus perceived of “*harmony*” only in the relation between the individual organism and its own *Umwelt* but not between the organisms of a certain place or “biocoenosis”. He states:

Von Harmonie ist im ökologischen Bereich allein bei der Ökologischen Nische, in ihrem Widerspiel von Organismus und Umwelt, zu sprechen. In den Beziehungen verschiedener Arten untereinander gibt es weder Harmonien noch Disharmonien. Was wir so auffassen, ist aus unserem Denken, noch dazu vielleicht aus unserer Ästhetik, hineingesehen. (p. 298f)

To speak of harmony in the realm of ecology is only possible with respect to the ecological niche, in its interplay between organism and *Umwelt.* In the relations between different species neither harmonies nor disharmonies exist. What we consider to be such is interpreted into it from our thinking, perhaps even from our aesthetics.

His conclusion from all this is thus scathing:

Aus allem Gesagten ergibt sich die Schlußfolgerung, daß, bezogen auf welche Raumeinheit immer, nicht ‘alles mit allem im Miteinander, Füreinander und Gegeneinander’ zusammenhängt, daß also *die Biozönose selbst und alle ihr unterstellten Eigenschaften und Fähigkeiten nur Gebilde des menschlichen Vorstellungsvermögens, daß sie eine Fiktion sind. Die Biozönologie als Wissenschaft hat keinen realen Grund*. (p. 300)

From all that has been stated here the conclusion arises that, related to any given unit of space, not ‘everything is connected to everything in a mode of with, for and against each other’, *i.e. that the biocoenosis itself and all the characteristics and abilities ascribed to it are merely products of the human imagination, that they are a fiction. Biocoenology as a science has no real basis*.^37^

Peus’ approach to the (animal) community can thus be considered a radically “individualistic” one, even more radical than that which Henry Allan Gleason (1917, 1926, 1939) developed for the plant community.^38^ While Gleason, at least in earlier versions of his “individualistic concept” (of the plant association), acknowledged that biotic interactions between different plant individuals and species populations led to a change in the conditions of the habitat (e.g. by shading), Peus did not consider such phenomena as a legitimate subject of ecological studies. Like Peus, however, Gleason did not speak of *interactions* but rather subsumed biological interactions of the kind described within the expression “environmental control” (Gleason 1917, p. 472).^39^ Peus even rejects the possibility of a discipline, of a science of bioconoeses (or even communities in the broader sense), seeing only autecology as “real” ecology. The biocoenosis for him neither had an ontological reality nor was it of any epistemological or methodological relevance (the latter of which at least Gleason and even Bodenheimer were willing to accept to some degree^40^; see also below).

In comparison, then, Peus was clearly more radical in his critique than Bodenheimer. He was also more systematic in his seminal paper from 1954, elaborating his arguments specifically against the biocoenosis concept, compared to Bodenheimer’s discussions of various community concepts beyond the biocoenosis. At the same time, Bodenheimer’s objection to holistic thinking was based on specific and explicit philosophical and epistemological foundations, while Peus did not disclose his thinking on these issues. Many of the two scientists’ arguments converged, however, such as the critique of the assumed “harmony of nature” and their focus on the individual species in contrast to the whole community.

## Saving the biocoenosis? Reactions to Bodenheimer’s and Peus’ critiques and the (re-)positioning of the biocoenosis concept

Peus’ text in particular drew a number of responses, but Bodenheimer’s critique also prompted some pointed critiques from German-speaking ecologists, such as Friederichs (e.g. in Schwerdtfeger et al. 1960/61 and Friederichs 1957a, 1967) and Thienemann (1956, referring to Peus only). Peus’ text and the question of the “nature” of the biocoenosis even became a major part of a colloquium. I will use the report of that colloquium to “crosscheck” the understanding of the biocoenosis held by German-speaking ecologists against the background of the critique voiced by Peus, Bodenheimer, and others.

### The 1959 “Colloquium on Biocoenosis Questions”

The colloquium was held in 1959 on the occasion of the 15th Annual Meeting of the German Society for Applied Entomology (*Deutsche Gesellschaft für Angewandte Entomologie*), which took place in Freiburg/Breisgau, its proceedings being published in 1960/1961 (Schwerdtfeger et al. 1960/1961). It is these proceedings to which I mainly refer in the following. The motivation for convening the “Kolloquium über Biozönose-Fragen” (Colloquium on biocoenosis questions) had been a presentation by Karl Friederichs during an earlier meeting (1957) entitled “*Bestehen in Kulturbiotopen Lebensgemeinschaften?*” (“Are there biocoenoses in cultural biotopes?”; see Friederichs 1957b). The colloquium dealt with three major questions, the third of which was explicitly sparked by Peus’ paper. These questions were (Schwerdtfeger et al. 1960/61, p. 90):

“How should the biocoenosis be delimited conceptually?”

“How can the biocoenosis be subdivided?”

“Is the biocoenosis a reality or a fiction?”^41^

As Schwerdtfeger^42^ states in his introduction to the colloquium, Peus had been invited to take part in the discussion but could not attend. Among the discussants at the colloquium were some of the most prominent German-speaking ecologists.^43^At least one of the speakers (Wolfgang Schwenke) also complained that the colloquium was too short (one-and-a-half hours!) to solve the “crisis” in which he considered biocoenology to be, and that it was too biased towards zoologists (in terms of the participants).

The most prominent speaker was certainly Karl Friederichs (Göttingen), whose closing words also constitute the longest text in the proceedings. While Wilhelm Kühnelt (1905–1988)^44^ was already a well-known zoology professor in Vienna at the time of the colloquium (since 1952), and the Hungarian János Balogh (1913–2002), teaching in Budapest, had already written an influential book on the theory of biocoenoses (or rather zoocoenoses),^45^ some of the speakers were still in the earlier stages of their careers. Joachim Illies (1925–1982)^46^ had just completed his habilitation a few years previously and was lead scientist at the small limnological river station of the Max Planck Society in Schlitz in Hesse, while in 1959 Wolfgang Schwenke (1921–2006) still occupied an insecure position at the University of Munich, not receiving a full professorship for applied zoology there until 1966.^47^

In order to compare their positions with that of Peus (and Bodenheimer), I have analysed the different contributions to the discussion and juxtaposed them with Peus’ main points of critique in terms of the nature or the existence of the biocoenosis (summarised in Table 1).

**Table 1:** Positions expounded by the main commentators in the 1959 “Colloquium on biocoenosis questions” (as extracted from the publication by Schwerdtfeger et al. 1960/61) with respect to the critique of the biocoenosis concept by Peus (1954)

	Kühnelt	Illies	Balogh	Schwenke	Friederichs
Existence	Yes	Yes	Yes	Yes, but…	Yes
Closedness	Not necessarily	−	−	−	Not really
Community	Yes, but…	−	Yes	−	Yes
Equilibrium	Yes	(Yes)	Yes	Yes	Yes
Self-regulation	Yes	−	Yes	Unclear	Yes
Unity/harmony	No	−	(Yes)	−	Yes
Superorganism	No	−	−	−	No

“−” means that the respective author did not touch on this point in his comment. See text

I have divided Peus’ “equilibrium” criterion into “equilibrium” and “self-regulation”, doing so for two reasons: first, as I will show, not all interlocutors present at the workshop regarded equilibrium and self-regulation as equivalent concepts and, second, the two concepts are not the same from a philosophical perspective either (see below). In addition, I also address the question as to whether the biocoenosis is said to be a “superorganism”, which Bodenheimer sees as the understanding of the concept held by most German ecologists, and which Peus touches on in relation to the issue of “unity”, especially with quotes from Thienemann. As can be seen in the table, not every author referred to all of these criteria, with the exception of Kühnelt and Friederichs.

To begin, then, all the authors agree that the biocoenosis “exists”, that it is a reality and not a fiction. As I seek to demonstrate below, however, the central issue of the whole debate is not only whether the biocoenosis exists per se but what “to exist” means to the different protagonists in relation to the question of the very “nature” of the biocoenosis. The discussion here reveals different ontological, epistemological and methodological perspectives and—to the extent that the biocoenosis is taken to be a holistic unit—different ideas of what “holistic” means.

If the biocoenosis exists, is it also a kind of superorganism? None of the participants in the Colloquium subscribed to this view, while Kühnelt and Friederichs explicitly rejected the notion. Friederichs, whose actual descriptions of the properties of the biocoenosis (or the holocoen) bore the closest similarity to the idea of a superorganism (see Jax 1998), in fact had never used this image but regarded the comparison to an organism as a useful heuristic principle at best (Friederichs 1927, p. 156). Instead, he emphasized as far back as 1927 that he considered the “higher units of life” (“*höhere Lebenseinheiten*”) to be what he called “organisations”, which he introduced in the following way: “An organisation is a biological whole that maintains its own existence by means of self-regulation” (“Eine Organisation ist eine biologische Ganzheit, die sich durch Selbstregulation bei Bestand erhält.”) (ibid). Thus the allegations levelled by Peus and Bodenheimer on this issue were refuted, even though some of the writings of German ecologists, especially Thienemann, actually gave cause for airing this allegation (see also Sect. 2, above).^48^

### Does the biocoenosis “exist”?

It is interesting now to see which arguments (and also empirical evidence) were put forward for the “*existence*” of the biocoenosis. While some authors, e.g. Joachim Illies, tried to prove the “objective” existence of the biocoenosis based on the results of their empirical studies (see below), for other authors this existence was the *starting point* of their argument and not the result of some conceptual and/or empirical discussion.

Thus, Balogh states that the biocoenosis is “a natural entity existing independently of human thinking” (“eine vom menschlichen Denken unabhängig existierende natürliche Einheit” Schwerdtfeger et al. 1960/61, p. 101)—which he describes as a consensus position shared by almost all (German) ecologists. He continues: “That the living community [i.e. biocoenosis] constitutes more than the sum of these effects [i.e. which the populations living near each other and the inanimate environment produce] is, however, *evident*.”^49^ Likewise, Friederichs in his—as often—patronising manner reiterates earlier statements of his own saying: “But the biocoenosis is no fiction, it is a very real fact” and “Actually, we almost all know it, but it has to be repeated over and over because it makes no sense to some people. *Anyone who does not accept the biocoenosis as a reality does not see the wood for the trees* […] One could also say instead: they hear only notes and not the melody”^50^ (alluding thus to his perception of Gestalt theory; see Friederichs 1937; Jax 1998). Friederichs specifically mentions Bodenheimer as one of those who are “unmusical” in this sense^51^ .

Illies tries to prove the existence of the biocoenosis by demonstrating that he was able to find three distinct, spatially clearly delimited sets of species (biocoenoses for him) following each other within the course of a stream, even though the physical and chemical parameters along this course formed a gradient without clear discontinuities. From this, he concluded that biocoenoses are “real phenomena of nature” (Schwerdtfeger et al. 1960/61, p. 99) and that “objective boundaries” of the same can be found.

### Further criteria that provide evidence for the existence of biocoenoses and/or characterise their “nature”

Concerning the specific criteria that Peus puts forward against the existence of the biocoenosis, a closer look reveals that there is at least some heterogeneity in the positions of the “German” ecologists, even though Illies and Schwenke deal almost exclusively with the question of the “existence” of the biocoenosis and its equilibrium. It is Friederichs, Balogh and, to some extent, Kühnelt who strongly defend almost all the properties of the biocoenosis postulated and criticized by Peus.

The only real concession Friederichs makes to Peus is the question of the *closedness* of the biocoenosis, on which he remarks: “No living collective is completely autarchic” (“Völlig autark ist kein Lebenskollektiv”) (Schwerdtfeger et al. 1960/61, p. 111). Kühnelt also acknowledges this point, stating that *not all* biocoenoses are autarchic.

It is the remaining criteria that can provide more insight into how the concept of the biocoenosis was perceived as “holistic” in German-speaking ecology by the late 1950s/early 1960s. These criteria also serve to illuminate the question of what is meant by the concordant statements that the biocoenosis really “exists”. What we encounter here, however, is considerable (although implicit) confusion about what the terms “community” (“*Gemeinschaft*”, in the social sense), “equilibrium”, “self-regulation”, “unity” and/or “harmony” mean.

Regarding the “*community*” and Peus’ question as to whether the relation of species in the community was one of “with each other, for each other, against each other”, Balogh and Friederichs remain rather vague (and emphatic, almost melodramatic).They nevertheless emphasize that species and populations are closely bound together with each other and with the environment (“by an unbreakable chain”, Balogh,^52^ “not imaginable as existing for itself but only in the whole of its biocoenotic nexus”, Friederichs.^53^ This corresponds to their a priori notion of an ontologically given existence of the biocoenosis (see also Sect. 2, above). Only Kühnelt is more specific in supporting the idea of such a community of closely linked species, albeit with the caveat that the mutual links between species are not regarded as strictly obligatory, as in a symbiosis. He even interprets Möbius (whose definition of the biocoenosis is his basic reference) as having understood “*Gemeinschaft*” (community) “in the sense of a recurrent common occurrence”^54^ of species, i.e. in a very weak sense, which comes closer to Petersen’s idea of communities. He also emphasizes that the parts of the biocoenosis are, to some extent at least, interchangeable—in the case of the oyster bed even the oyster itself (!) can be substituted if it has been wiped out by another species.^55^

The discussions as to whether “*equilibrium*” and *“self*-*regulation”* respectively are properties (or even defining criteria) of the biocoenosis are closely linked for most authors. Kühnelt refers to Möbius’ “biocoenotic equilibrium” not as a static state but as “fluctuations around average values” (“*Schwanken um Mittelwerte*”) (ibid. p. 92) (but fluctuations of which factors?). Balogh and Schwenke seem to take the mere continued existence of a community (Balogh uses the example of a beech forest) as proof of a kind of equilibrium and self-regulation. Friederichs emphasizes that the biocoenosis has a kind of steady state or dynamic equilibrium (thus allowing also for succession phenomena^56^). Friederichs’ idea of a biocoenotic equilibrium is also cited (without further comment) by Illies. For some authors, e.g. Balogh and Friederichs, equilibrium is the same as or indeed almost *evidence of* self-regulation,^57^ while others distinguish the maintenance of an equilibrium qua *self*-*maintenance* (*Selbsterhaltung*)—which might also be explained by autecological factors—from *self*-*regulation;* the latter is seen as an “emergent” property of the biocoenosis, on account of the strong interrelations between its parts.^58^ Kühnelt sees self-regulation as an empirically supported part of Möbius’ original definition, but he provides no further proof of this assertion other than that self-regulation appears to be evidenced for him by the persistence of the “biocoenotic equilibrium”. This inference from perceived pattern (equilibrium or constancy; see below) to underlying mechanisms is a fairly popular figure and constitutes a major argument in corroborating the existence of the biocoenosis as a self-regulated entity.

The issue of “*harmony*” and “*unity”* is taken up explicitly and affirmatively only by Friederichs. Kühnelt speaks of the biocoenosis as a “false whole” (“*unechte Ganzheit*”) or a “ lower-order whole” (“*Ganzheit niederer Ordnung*”) (ibid. p. 94) because, as mentioned before, he considers the components of biocoenosis to be interchangeable to a considerable degree without its “overall character” (“*Gesamtcharakter*”)^59^ being compromised. In Friederichs’ earlier publications especially, however, the “harmony” and “unity” of the biocoenosis (or, if not of the biocoenosis then at least of nature as whole) is shown to be a central tenet of his fundamental philosophy^60^, a point which prompted especially vehement critique from Bodenheimer and Peus.

### Excursus: German ecologists and holism during the National Socialist era

Before moving on to the discussion, I want to touch on another historical context that contributed toward shaping the controversy (or the tone in which it was conducted), at least as it was conducted between some of the opponents of the controversy described above. The question arises as to what role political and ideological positions played, especially in the discourse between Bodenheimer and the mainstream German ecologists, and in particular regarding the role of German ecologists during the Nazi era. An indication of this can be found in the rather sharp and almost personally insulting comments exchanged between Friederichs and Bodenheimer in their publications, mostly in footnotes (e.g. Bodenheimer 1958, p. 165, FN; Friederichs 1957a, p. 136 and FN 31 therein). Even though I have not undertaken any in-depth biographical research in the archives, and the reasons thus remain somewhat speculative, it can be taken for granted that, apart from holding differing scientific opinions, their highly different political positions during the period of German National Socialism also played a significant role in this overtly personal animosity, given that Bodenheimer was a staunch Zionist and Friederichs a person who, at the very least, tried to profit from the Nazis. Bodenheimer, for example, wrote about Friederichs: “K. Friederichs, who mottoed [sic!] each chapter of his ‘Ökologie als Wissenschaft von der Natur’ (1937) with a sentence taken from the fundamental ecological book of Adolf Hitler ‘Mein Kampf’ is the extreme exponent of the German school.” (Bodenheimer 1958, p. 165, FN).^61^ Along with many German ecologists, Friederichs clearly leaned towards the right wing of the political spectrum, to say the least. In contrast to German physicists, for example, there was no marked brain drain (emigration from Germany) by biologists—or more specifically ecologists—during the Nazi area (see Deichmann 1995 for an overview). Neither Friederichs nor Thienemann were regular members of the National Socialist party but they were far from being opposed to it; Friederichs was even member of the National Socialist Teachers League and the “*Opferring*” of the NSDAP (Buddrus and Fritzlar 2007, p. 135). In the booklet to which Bodenheimer refers, Friederichs (1937) does not in fact preface any of his chapters with a motto or quote from Hitler, in contrast to the former’s claim. However in at least three places, he tries to curry favour with the Nazis, in one place indeed using a literary quote from Hitler (Friederichs 1937, p. 86), and in two others by advertising for ecology (which had no real place at German universities yet) as being the science of “blood and soil”: “If we, though not claiming originality but understandably can call ecology the *doctrine of ‘blood and soil*’…” (ibid, p. 91).^62^ Similar statements that sought to attract support for the importance of ecology within the new NS state (largely unsuccessfully), can also be found in Thienemann. As Potthast (2006, p. 380)^63^ writes, although Thienemann was no National Socialist, he did not object to some of his close assistants (e.g. Erich Wasmund) openly displaying their active support for the Nazis. Thus it is not surprising that there was strong political and ideological disagreement between Bodenheimer and the (for the most part) highly conservative German mainstream ecologists—even more so after the horror of the Holocaust came to light. I do not think, however, that this was a major reason for the scientific and philosophical differences they had with respect to the nature of the biocoenosis. First, Bodenheimer had already developed his ideas about the density-independent regulation of insect populations (de-emphasizing the role of other species such as parasites and predators and thus the importance of the community) before the Nazi dictatorship, his first publications appearing between 1925 and 1928. His theoretical approaches and his epistemological position at that time (arguing that theory should be built on mechanistic principles) were already in line with his later mode of argumentation and did not change substantially afterwards. It appears (if we follow Harpaz 1984) that his empirical studies in Israel shaped his view of population regulation, especially observations on the effects of catastrophic climatic events on insects. It is thus unlikely that the point of departure for his theoretical stance was a political one. Second, I could find no evidence that the position adopted by Peus, whose career continued largely unimpeded during the 1930s and WW2 (Schumann 1980), was in political opposition to the majority of his German colleagues.

Another question might be why holistic concepts in German ecology persisted up to the late 1960s, in spite of the fact that holism, at least in some fields, had become largely discredited by its (mis-)use during the Nazi era (Harrington 1996; Gilbert and Sarkar 2000). In fact, as has been noted before (Jax 1998; Potthast 2006), there was no substantial change in the ideas of the most prominent German ecologists after the war. Thienemann only deleted a few sentences which had referred too directly to Nazi ideology in one of his older texts before it was republished (see Potthast 2006). He also remained director of his institute after the war. Although Friederichs retired in 1945 (at the age of 67), he was still awarded honorary membership by several (German) scientific societies after the war (Schimitschek 1970; Universität Rostock 2018). So there was continuity within German ecology, and the influence of holistic thinking went largely uninterrupted until the late 1960s. I have argued in an earlier paper (Jax 1998) that this might also have been one reason for the rather late arrival of ecosystem research in Germany, but it remains a somewhat open question why holistic ideas in postwar German ecology did not come under critique as being too closely related to Nazi ideology. What can be said is that after the death of both Thienemann and Friederichs in the 1960s, much of the specific philosophical underpinnings on which they based their arguments gradually went on the wane in German ecology. Although some “holistic” thinking—usually rather vague^64^—persists in ecology (also outside Germany) and even more in ecology’s public perception, today’s research practice is for the most part highly pragmatic and lacking any marked ideology.

## Discussion

The divergence of opinions regarding the existence and nature of the biocoenosis between Peus and Bodenheimer on the hand and mainstream German ecology (as manifested here especially by those discussed above) on the other points to a range of fundamental philosophical issues. Some of these have already been touched upon above in analysing the debate on the paper by Peus. I will now situate these philosophical issues within the larger philosophical debate on the “nature” of ecological units, especially concerning the relation between the ontological, epistemic, and methodological status of the same.

The first issue is the ontological status of ecological units, here specifically the biocoenosis. Especially for Peus, who adopts the most radical position, it is the individual organism alone rather than assemblages of organisms that possesses a legitimate ontological status. At the other extreme, Friederichs starts from the assumption of the ontological existence of integrated wholes and thus pursues an ontological holism. Kühnelt takes a somewhat intermediate position: his idea of the biocoenosis does not fit—to use Odenbaugh’s terminology (2006, p. 216)—the notion of an “individual” (as highly structured, interacting and integrated) but rather that of a “whole” (with some causal relations existing between the parts); he would allow many changes in species composition while still seeing the “overall character” (“*Gesamtcharakter*”) of the biocoenosis maintained. Friederichs and Balogh tend towards the “individual” end of that gradient whereas Peus and Bodenheimer would consider biocoenosis (if, as with Peus, they would want to use the term at all) much more as possessing the nature of an “aggregate” (with few or no causal relations among the parts; Odenbaugh, op.cit).

The other question is the epistemic and methodological status of the biocoenosis. It appears that most authors, even Bodenheimer (though not Peus), admitted that the biocoenosis may be said to exist, or could be conceived of at least in terms of a useful epistemic object.^65^ This means that (recurrent) and spatially delimitable co-occurrences of species (like Petersen’s communities) could be useful objects and starting points for ecological investigations. Bodenheimer even stated: “We gladly accept thus the supraorganismic community concept as a valuable heuristic principle but we refute its claim to be an established fact” (Bodenheimer 1957, p. 87). This, however, does not necessarily go hand in hand with supporting a methodological holism (Bergandi 2011). Friederichs, for example, emphasized time and again (explicitly against Bodenheimer) that the biocoenosis—and the holocoen—were “completely available to rational analysis” (“völlig rational analysierbar”, Schwerdtfeger et al. 1960/61, p. 112; “a system entirely open to analysis” “ein restlos analysierbares System”, Friederichs 1957a, p. 136) and did not have any “mystic” properties. Friederichs’ ontological holism did not prevent him from using a pragmatic classical approach to investigating biocoenoses and holocoens, which otherwise would have resulted in a problematic circle of first having to know the whole before being able to understand the parts and vice versa (see Phillips 1970; Jax 1998). Thus Friederichs (1930, p. 114) wrote: “[T]he method of investigating single processes and states of life is not influenced substantially by the holocoen perspective; the method remains the same as with the isolating observation of nature. However, many riddles may be unveiled by the former that would have forever remained a riddle to the latter”^66^ (for more details see Jax 1998 p. 133ff; Potthast 2020).

The mixture between an apparently pragmatic methodological approach towards the biocoenosis or other “higher units of life “ and its connection to ontological holism obviously caused a problem for many critics, one that they may not have perceived as such at all but simply viewed under the (undifferentiated) heading of “mystic” holism and organicism. In fact the ontological holism propagated by, for example, Friederichs and Thienemann was connected to an underlying philosophy that, in keeping with the trend of the early 20th century, emphasized that the “evident” unity and wholeness of nature was, to a considerable degree, also a matter of intuition (e.g. Friederichs 1957a, p. 139) and a kind of “viewing” (“*anschauende*”) perspective in the sense of the contemplation of nature (e.g. Thienemann 1954, p. 317ff; see also Jax 1998 and Sect. 2, above). The allegations made by Bodenheimer and Peus are thus understandable if the philosophical writings of German mainstream ecologists were taken seriously at that time or indeed are taken seriously today.

Another problem highlighted by the critics of the biocoenosis concept and backed up with good arguments is the question of how to infer the actual properties of a biocoenosis from its delimitation (or identification, depending on one’s ontological position), in other words: which of the (postulated) properties of the biocoenosis were taken for granted? In fact, based largely on the original definition of the biocoenosis by Möbius, a set of specific properties were often associated with *any* set of organisms labelled a biocoenosis, namely, those properties that Peus identified and rejected for not being valid properties of the biocoenosis. As discussed above (Sect. 4), equilibrium and self-regulation were often simply presumed or inferred from (recurring) species compositions at a site, if the species composition displayed a certain persistence over time. They were also seen as properties that corroborate the holistic, integrated nature of the biocoenosis. This problem has persisted in ecology, nowadays usually with reference to ecosystems: based on a spatial (often physiognomic) delimitation of an ecological unit, problematic inferences are made regarding the properties of such units, again especially their ability to self-regulate (see Jax 2007 for a detailed discussion of this issue). For Bodenheimer (and also Gleason) the task of the researcher was first to delimit an object from the whole of nature. This could be done and frequently is done in terms of recurrent species combinations and/or discrete boundaries in space, such as the boundary between forest and meadow, or between the oyster bed and the sea floor surrounding it. *Only then*, they insist, can one consider and investigate the specific properties of this object (such as self-regulation or close interactions between species) without inferring too much simply from the patterns observed.^67^ In consequence, *only some* of these objects (e.g. species assemblages) would be called “biocoenoses” according to the definition espoused by Möbius (and German mainstream ecology of that time), while others would not.^68^ A broad range of terminology has developed to account additionally for “imperfect” biocoenoses, e.g. merocoenoses (*Merozönosen*) or subtypes of biocoenoses (see e.g. Balogh 1958, p. 41ff; Schwerdtfeger 1975, p. 15ff). However, the terminology of biocoenoses was itself rather sloppy in most cases, including the common practice of calling (any) forest or meadow, for example, a biocoenosis (and later an ecosystem), without first investigating it to see if it had the postulated properties of the biocoenosis (or ecosystem).

Such issues did not completely escape German (mainstream) ecologists. Thus, for example, in the “Colloquium on biocoenosis questions” Fritz Schwerdtfeger questioned the inference that Illies made, namely, taking discrete patterns of species composition in a stream gradient as proof of the existence of communities. Schwerdtfeger, like Peus, saw that this pattern might also be a result of autecological factors (p. 109), while Bombosch stated that a constancy of species composition and even constancy in their relative abundances were not necessarily proof of self-regulation (as stated by others).

These critiques, however, as well as those of Bodenheimer and Peus, did not initially lead to a more critical reading of the biocoenosis concept overall. In my experience, it was only with the stronger orientation towards English-speaking ecology, and English as the main and almost only language for scientific publications in ecology, that the dominance of the biocoenosis concept in the form described above waned in German-speaking ecology. The term was replaced on the one hand by the more flexible and pragmatic community concept and on the other by the “ecosystem” concept, which was increasingly used as the central ecological unit. Some of the old problems have surfaced again, however, at least for the ecosystem, as described above (and see Jax 2007). And in terms of ecological communities, the 1990s saw a new debate in vegetation science on the question as to whether (plant) communities (or even vegetation science) “do exist” (Wilson 1991, 1994; Keddy 1993; Palmer and White 1994 and others), a debate plagued by a similar lack of clarity as that discussed in this section.

## Conclusions

The discourse on the existence and nature of the biocoenosis is an almost forgotten one, barely known of at all outside German-speaking countries. Yet it is of interest not least because it parallels some familiar debates in the United States, namely, the holism-reductionism debate in the context of the—now “classical”—juxtaposition between the approaches of Clements and Gleason (e.g. Tobey 1981; McIntosh 1985; Odenbaugh 2006). In contrast to the American debate and the community concept, the concept of the biocoenosis has a clear historical origin as a technical term not used in everyday language, having been defined by Möbius in 1877. Another difference is that the major protagonists in the Anglo-Saxon realm were almost all botanists, while the discourse on the biocoenosis was, from its very beginnings, heavily dominated by zoologists (a circumstance about which some participants in the 1959 colloquium on biocoenosis questions also complained).^69^

The critique by Bodenheimer, in spite of its sometimes sharp tone, was comparatively moderate in substance, acknowledging at least the usefulness of some idea of biocoenosis (or, as he saw it, a more general ecological community), while Peus’ paper (which was not followed by any other papers of the kind by him) and the “individualistic” approach described therein was even more radical than that of Gleason. The critique, especially that of Peus, helps to sharpen our view of the German mainstream position, precisely *because* it was so radical and practically forced the proponents of the “classical” biocoenosis concept to respond quite specifically and to lay out their arguments again.

It is clear from this (and other writings cited, for example, by the later Thienemann) that the term “superorganism” for the biocoenosis, or even a close comparison between the two, was rejected even by leading “holistic” German ecologists by the 1950s/1960s. Thus if, as is often said, their approach was an “organismic” one, then it was so only insofar as “holism” and “organicism” were frequently used synonymously, including in the early 20th century (e.g. Alverdes 1936, following Adolf Meyer-Abich, Gilbert and Sarkar 2000)—which may have been misleading in this case.^70^ Even when called “holistic” in the sense of merely emphasizing the interrelations within a community and the importance—or even dominance—of the “whole” in contrast to the parts, an ontological holism did not normally coincide with a methodological holism. Bodenheimer and Peus certainly started out from completely different ontological and epistemological assumptions than their mainstream opponents. While they were explicitly following a rationalistic and even empiricist view of scientific endeavour, the underlying philosophy of, for example, Friederichs and Thienemann owed much to German idealism and romantic philosophy, as described above.

The most important difference between the opponents, in my opinion, was that Bodenheimer and, even more so, Peus put their finger on a number of thorny methodological issues which were readily ignored by the mainstream ecologists or were only addressed in a much more cautious way (e.g. Caspers 1950; Schwenke 1953), without really endangering the established consensus. Bodenheimer and Peus may have overshot the mark with some aspects of their critique, but they certainly hit the spot with their allegation that biocoenology (then) was often based on too many speculative elements. In this regard they certainly deserve to be given a hearing—even today.

## Footnotes

1 In the title as well as in the text I am referring not only to ecology in Germany but to ecology in the German-speaking world more broadly. In early ecology and up until WW2, German was widely used in scientific publications on ecology in Europe; it was not restricted to Germany, Austria and Switzerland but was also frequently used in France, Scandinavia, and even Russia (see also Schwarz and Jax 2011). Thus when, for the sake of brevity, I use the expression "German ecology" in the text, I am referring to this broader research community.

2 Jax (2006, p. 239): "In order to provide a formal definition, ecological units will be understood here as all those units that are subject to ecological research and comprise more than one single organism".

3 "Die Wissenschaft kennt noch kein Wort für eine solche Gemeinschaft von lebenden Wesen, für eine den durchschnittlichen äusseren Lebensverhältnissen entsprechende Auswahl und Zahl von Arten und Individuen, welche sich gegenseitig bedingen und durch Fortpflanzung in einem abgemessenen Gebiete dauernd erhalten. Ich nenne eine solche Gemeinschaft Biocoenosis oder Lebensgemeinde." (Möbius 1877, p. 75f); note that the English quote is taken from the translation of Möbius’ study by H. J. Rice (Möbius 1883). I have indicated where the text of the translation deviates from the original in respects relevant to the current study.

4 The concept was most popular among animal ecologists and aquatic ecologists, much less among plant ecologists, whose main ecological units were the (plant) association and the formation, the German speaking research community being dominated by plant sociology (see Whittaker 1962). Only a few plant ecologists, such as Helmut Gams in Austria and G. Einar Du Rietz in Sweden, made extended use of the biocoenosis concept—albeit in very specific interpretations (see Jax 2002).

5 George Evelyn Hutchinson discussed the biocoenosis concept in Vol. 2 of his "Treatise on Limnology" (Hutchinson 1967, p. 227f). He rejected the usage of complex definitions of the concept, as established in the German literature, because it appeared to him as too ambitious to apply in the field. He opted instead for a rather generic definition of the biocoenosis as "the totality of organisms living in a biotope, or the living part of an ecosystem" and continued: "All questions of interdependence, other than the possibility of its occurrence, self-regulation, and progressive maturation or succession are regarded as empirical, not deductively answerable from the definitions." (p. 230).

6 This section draws heavily on an earlier paper of mine (Jax 1998) which discusses another important "organismic" concept in German ecology, namely the "holocoen" of Karl Friederichs (and related concepts).

7 "In den seit Erscheinen der Arbeit von Möbius verstrichenen acht Jahrzehnten ist der Möbiussche Begriff der Biozönose im wesentlichen unverändert geblieben, obwohl sich in dieser Zeit unsere Kenntnisse über die Biozönosen erheblich erweiterten und obwohl sich eine ausgiebige—manchmal vielleicht etwas spekulativ geartete—auf's Theoretische gerichtete Aktivität entfaltete." See Sect. 4 for some biographical details on Balogh.

8 There are many different ideas and movements that have been located beneath the headings of "holism" and "organicism". In the heyday of these approaches, i.e. in the early 20th century, the terms "holism" and "organicism" were generally used as synonyms (see Needham 1928; Phillips 1970; Gilbert and Sarkar 2000). A clear delimitation of either concept has only rarely been undertaken, even to this day. I will also use the two terms largely synonymously, and only stress the "organicist" one when making an explicit comparison or equating the biocoenosis (or the community) with an individual organism.

9 A detailed account of the roots of holism in Germany (with special reference to psychology and biology but none to ecology) and their influence on German culture is given by Harrington (1996).

10 August Thienemann was one of the most prominent German ecologists. He was, among other things, a co-founder (with Einar Naumann) of the International Society for Limnology as well as the long-time director of the Max Planck Institute for Limnology (known up until 1948 as the "Hydrobiological Institute of the Kaiser-Wilhelm Society") in Plön, northern Germany. See Schwabe and Brundin (1961), also Schwarz and Jax (2011) and Potthast (2020).

11 Karl Friederichs was an applied zoologist (entomologist), well known for his two-volume book on the foundations of applied zoology (Friederichs 1930) but even more for his theoretical work. For much of his career he taught and worked at the University of Rostock. In 1941 (at the age of 63) he became full professor of applied zoology at the new (and short-lived) "Reichsuniversität Posen", established by the Nazis in the then occupied Polish town of Poznan (Schimitschek 1970; Buddrus and Fritzlar 2007; Universität Rostock 2018; see also Potthast 2020).

12 In the US the most influential "organicist" was the plant ecologist Frederic Clements. As his theory of the "plant formation as an organism" is rather well-known and has been widely discussed (e.g. Tobey 1981; Kingsland 2005; Eliot 2007), I will not cover this in detail here.

13 Friederichs refers explicitly to the concept as used by Wolfgang Köhler, e.g. in Friederichs (1937), p. 6; see also Jax (1998).

14 "…und wir müßten blind sein, um nicht zu erkennen, daß alles aufeinander geordnet und aufeinander angewiesen ist”.

15 "So wie die Welt ein dynamisches System ist, das sich durch Selbstregulierung in einem labilen Gleichgewichtszustand erhält, so gilt das gleiche von natürlich abgegrenzten Abschnitten der Biosphäre (z.B. Teich, Moor, Strand)".

16 "Die Darstellung der ‚Lebenseinheiten‘, wie ich sie oben gegeben habe—ihre ‚Entwicklung‘ vom Einzelwesen über Lebensgemeinschaft zum Holocoen—diese ganzheitliche Auffassung der Natur ist zweifellos eine ‚anschauende‘ Naturbetrachtung morphologischer Art…".

17 For other critical statements about the "nature" of the biocoenosis and at least some of its postulated properties, see e.g. Caspers(1950), Schwenke (1953).

18 Friedrich Bodenheimer's father Max Bodenheimer, together with Theodor Herzl, was one of the co-founders of the World Zionist Organisation (Bodenheimer 1959; Uvarov 1959; Harpaz 1984; see also there for other biographical details on Friedrich Bodenheimer).

19 This publication appeared in Bodenheimer’s native language, German. With the rise of the Nazis in Germany, he ceased publishing in German, all his later publications being in English, or occasionally in French and Hebrew (Harpaz 1984, p. 21; Böhme 2018).

20 "Wegen dieser Grundeinstellung schon werden die dargelegten Prinzipien den lebhaften Widerspruch der heutigen Biozönotiker hervorrufen, die bereits erklären, daß der genius loci oder der biologische Einheitsfaktor mehr ist als die Summe aller einzelnen bewirkenden Faktoren einer ökologischen Einheit." A footnote at the end of this sentence refers to Friederichs' paper from 1927.

21 E.g. a four-page section entitled "Epistemological approach" in Bodenheimer 1957. Here, and in Bodenheimer 1958, he discusses Kant and Bertrand Russell in particular.

22 This comparison of understanding the biocoenosis (or nature as a whole) to listening to music was a recurrent theme of Friederichs; see below. Here, then, Bodenheimer also positions himself clearly against what has been described above as a "viewing" approach towards nature (a similar quote can be found in Bodenheimer 1938, p. 142).

23 In this work he dealt with the issue of the nature of the community, especially in the chapters "Is the animal community a dynamic or a statistical conception?" (1938) and "Is the animal community a dynamic or merely a descriptive conception?" (1958) respectively (note the addition of the word "merely" in the 1958 version).

24 The position and the arguments of Bodenheimer did not change much over the decades, and parts of the text are identical in the different papers.

25 He there wrote: "It was not long after my arrival in Palestine that I became convinced that weather factors are much more important than parasites in the control of insect species".

26 Similar arguments were put forward by other scientists, as described in the discussion in Sect. 4.

27 While Uexküll can himself be seen as an organicist or holistic biologist (see chapter 2 in Harrington 1996), he did not deal with ecology as such. If he did so implicitly, he did not go beyond what one would call "autecology". His focus was on the (harmonious) unity of the (individual) organism and its environment, the "*Umwelt*", but he never went beyond the single species as the relevant unit (though he did at times think of the *human state* as a functional unit). In other words, the biocoenosis—or other multi-species units—were something Uexküll never dealt with (Mildenberger and Herrmann 2014, p. 305). He also rejected the idea of the priority of the whole over the parts, as postulated, for example, by his contemporary Adolf Meyer-Abich. Karl Friederichs, who according to Mildenberger and Herrmann (2014) was sympathetic to Uexküll, explicitly regarded Uexküll's concept of "*Umwelt*" as not being useful for ecology, at least not beyond autecology (e.g. Friederichs 1943, p. 147).

28 Becoming an ecologist in the early 20th century was still not a common career path (in Germany as elsewhere). The first university chair explicitly dedicated to "ecology" in Germany was not established until 1963, in Kiel, being filled by Wolfgang Tischler. With regard to applied ecology, Harpaz (1984, p. 3) recounts a conversation between Richard Hesse and Bodenheimer: when the latter suggested to his adviser that he might write a zoological dissertation with an applied character, Hesse told him "that Bodenheimer could not expect him [Hesse] to take any interest in his work, since by choosing applied entomology as a profession Bodenheimer had given proof of his lack of genuine interest in science. Only in times of stress could a zoologist go over to applied zoology, said Hesse".

29 Some of the ideas elaborated in this paper had already been touched upon more in passing in an earlier publication (Peus 1951), one which does not seem to have attracted much attention.

30 He emphasizes, however, that his general considerations are also applicable to plant communities (Peus 1954, p. 272).

31 Bodenheimer (1958) twice refers affirmatively to Peus' paper, regarding the latter's "ökologische *Umwelt*" as close to his own concept of the "eco-world" (p. 165) and also sharing his critique of the biotope concept (p. 167).

32 In contrast to Bodenheimer, Peus does not mention Uexküll, although he makes much broader use of his ideas and terminology. It is beyond the scope of this paper to compare Uexküll's and Peus' use of terms in detail. On Uexküll's ideas and his concept of "Umwelt", see e.g. Brauckmann (2001), Kull (2001), Mildenberger and Herrmann (2014), Brentari (2015).

33 Which is, however, the most common meaning of "*Umwelt*" in German today: Umwelt = environment. This is to be distinguished from the technical term "*Umwelt*" as used by Uexküll and also by Peus.

34 Reswoy's paper was in fact written in Russian, with only a slightly extended summary in German. The main points stated in this summary were very well received, and the paper has frequently been cited since by German ecologists.

35 "Als wesentliche Merkmale des Biozönosebegriffes schälen wir aus den obigen Zitaten noch einmal die folgenden heraus: **Geschlossenheit**: Sättigung—Autarkie (Selbständigkeit, Unabhängigkeit); **Gemeinschaft (für die Glieder)**: Gegenseitige Bedingtheit—feste Bindung aneinander—Abhängigkeit voneinander—Miteinander, Füreinander, Gegeneinander—alles mit allem zusammenhängend; **Gleichgewicht**: Fähigkeit zur Selbstregulation, zur dauernden Selbsterhaltung, sich bei Bestand zu halten—relativ konstantes Mengenverhältnis der Arten und Individuen zueinander, ein Sich-die-Waage-Halten—Ausgleich zwischen Produktion, Konsum und Reduktion; **Einheit**: Harmonie—biologisches System—Organisation (‘Organismus höherer Ordnung’ ‚’organische Individualität höherer Stufe’)—Ganzheit—demgemäß: Lebewesen als ‘Glieder’, nicht Teile dieser Einheit.” (p. 295)

36 The wording alludes to the distinction between *Gemeinschaft* (community) and *Gesellschaft* (society) in the tradition of the sociologist Ferdinand Tönnies (1855–1936). Drawing on established uses of the terms in everyday German language, he established both of them as technical terms of sociology. Tönnies (1887, p. 5) wrote: "*Gemeinschaft* is the permanent and true living together, *Gesellschaft* only a transient and seeming one. And it is in accordance with this that *Gemeinschaft* should be understood as a living organism and *Gesellschaft* as a mechanical aggregate and artefact." ("Gemeinschaft ist das dauernde und echte Zusammenleben, Gesellschaft nur ein vorübergehendes und scheinbares. Und dem ist es gemäss, dass Gemeinschaft selber als ein lebendiger Organismus, Gesellschaft als ein mechanisches Aggregat und Artefact verstanden werden soll."). Examples of a *Gemeinschaft*, then, are marriage, family, or religious community, while trading companies or tourist parties are examples of a *Gesellschaft*. Interestingly, Tönnies also uses an organism metaphor for *Gemeinschaft* in the quote above. See e.g. Levine (1995) for the use of organism metaphors in sociology.

37 In a similar way, Bodenheimer (1958, p. 167) states that even though biocoenology could be a "potentially useful method", "bioceonology belong [sic!] in neither sense to ecology, but rather to philosophy or geography".

38 See Nicolson and McIntosh (2002) for a thorough and contextualising reading of Gleason's ideas that corrects some exaggerations and common misinterpretations of his individualistic concept.

39 I could not find any explicit references to Gleason, either in Peus' or in Bodenheimer's publications. The entire debate about the biocoenosis was conducted almost exclusively among zoologists and drew almost exclusively on zoological literature.

40 Gleason did not deny in general that something that can be called plant communities/associations "exists" in space. What interested him instead was what a plant community *is* (Gleason 1939, p. 93), i.e. the "nature" of the plant community, which he rejected as "organismic", as described especially by Frederic Clements (1916).

41 "'Wie ist die Biozönose begrifflich abzugrenzen?', 'Wie ist die Biozönose aufzugliedern?', 'Ist die Biozönose eine Realität oder eine Fiktion?'".

42 Fritz Schwerdtfeger (1905–1986) was a forest entomologist and population ecologist. At the time of the colloquium he was leader of the Lower Saxony Forest Research Institute (Niedersächsische Forstliche Versuchsanstalt) and in addition was teaching population ecology at the University of Göttingen. He later (1963–1975) also became well known for his 3-volume textbook on animal ecology.

43 There is no contribution by August Thienemann in the proceedings, but as Thienemann died in April 1960 it may be that he was not able to attend the meeting at all.

44 For biographical details see e.g. Schaller 1990.

45 Balogh (1958). The first edition of this book was published in 1953 as "A zoocönológia alapjai—Grundzüge der Zoozönologie" ("The fundamentals of zoocoenology"), with the Hungarian original and the German translation printed in one and the same volume. For biographical details, see e.g. Dózsa-Farkas (2003), Bakonyi (2003) and several other papers on Balogh in the same issue of that journal.

46 See Fittkau and Schmitz (1985) for more biographical details. In addition to his limnological and taxonomic work, Illies later also became known for his popular science work at the interface between biology, anthropology and theology: In parts of these writings he fervently argued—not least due to his specific interpretation of the Christian faith—against the Darwinian concept of evolution. In 1981, one year before his death, he was also one of the first signatories, along with 14 other German university professors, of the "Heidelberg Manifesto" which warned against "foreign infiltration" (*"Überfremdung"*) of German language and culture. The document, while applauded by the far right, was rejected by a much broader public who perceived it as an attempt to provide a "scientific" legitimation of racism and xenophobia.

47 In 1964, however, he became acting director of the university chair. In addition to his many publications on forest pests, he published two longer theoretical papers on biocoenology and applied entomology, in (1953) and (1955) respectively.

48 As stated above, Thienemann repeatedly mentioned in his writings that one could perceive of biocoenoses as "higher-order organisms". But he also emphasized (at least in his 1954 publication, p. 310) that a better word than "organism" might be (following Friederichs) "organisations" ("*Organisationen*"); see Potthast (2020).

49 "Daß die Lebensgemeinschaft mehr darstellt als die Summe dieser Wirkungen [d.h., welche die nebeneinander lebenden Populationen und das leblose Milieu produzieren] ist jedoch *offensichtlich*." (ibid, p. 102, my emphasis).

50 "Aber die Biozönose ist keine Fiktion, sie ist ein sehr realer Sachverhalt." (Schwerdtfeger et al. 1960/61, p. 110) and "Eigentlich wissen wir das fast alle, aber es muss immer wieder durchexerziert werden, weil es einigen nicht einleuchtet. *Wer die Biozönose nicht als Realität anerkennt, der sieht den Wald vor Bäumen nicht* […] Man könnte statt dessen auch sagen: nur Töne und nicht die Melodie hören." (ibid. p. 112). Similar: Friederichs 1957a, p. 136.

51 Bodenheimer himself used a similar image in an earlier paper (Bodenheimer 1957, p. 86; see above).

52 "mit einer unzerreißbaren Kette" (Schwerdtfeger et al.^1960^/61, p. 102).

53 "nicht für sich existierend denkbar sondern nur im Ganzen ihres biozönotischen Konnexes" (ibid. p. 110).

54 "im Sinne eines regelmäßigen, gemeinsamen Vorkommens" (ibid., p. 92).

55 For Kühnelt, this is one argument for why the notion of the "superorganism" as applied to the biocoenosis is not appropriate.

56 In contrast to the discussion in the Anglo-Saxon realm, e.g. Clements (1916), the successional dynamics of species assemblages in general did not play an important role in defining and characterising communities or biocoenoses in Germany and continental Europe (see also Whittaker 1962).

57 Self-regulation is part of Friederichs’ definition of an "organisation", which is how he sees the biocoenosis—in contrast to a "superorganism" (see above).

58 Contribution to the discussion by Siegfried Bombosch (*1925) in Schwerdtfeger et al. 1960/61, p. 108f.

59 This relates to the question of what I have called the "self-identity" of ecological units (Jax et al. 1998). How much change do we allow to occur in an ecological unit and still consider it to be the "same"?

60 Thus he writes in 1937: "Harmony is simply a precondition for the diversification of being, a logical postulate of its existence, of the is-ness of nature. If there were no harmony, all singular phenomena would destroy each other. It exists from the very outset (pre-stabilised) in the basic properties of the elements." "Harmonie ist einfach eine Voraussetzung der Mannigfaltigkeit des Seins, ein logisches Postulat ihres Bestehens, des Soseins der Natur. Bestände nicht die Harmonie, so müßten die Einzelerscheinungen einander zerstören. Sie ist von vornherein bestehend (prästabiliert) in den Grundeigenschaften der Elemente.” (Friederichs 1937, p. 41).

61 In remarkable contrast to this Bodenheimer, in his autobiography (1959, p. 362ff), on almost two full pages quotes a paper by Friederichs (1929), who reviewed Bodenheimer's 1928 publication on population regulation, and then comments on Friederichs' paper with the sentence: "There is little to add to this eminently fair review by an adversary." (p. 364). To my knowledge this the only place in the autobiography where Friederichs is mentioned.

62 "Wenn wir, mit Verzicht auf Originalität aber verständlich Ökologie als die *Lehre von ‚Blut und Boden*‘ bezeichnen können…".

63 See this publication for more details on the issue of German ecology and National Socialism.

64 Such an (often a-historical) "re"-discovery of holism has also been promoted by the environmental movement and "New Age" protagonists of the 1980s and 1990s (Gilbert and Sarkar 2000). On the different uses of "holistic" within contemporary ecology, see e.g. Wilson (1988).

65 See also the discussion about Gleason's approach (Nicolson and McIntosh 2002 and above).

66 "[…] wird die Methode der Untersuchung der einzelnen Lebensvorgänge und -zustände durch die holocöne Anschauung nicht wesentlich beeinflußt; die Methode bleibt die gleiche wie bei isolierender Naturbetrachtung. Manches Rätsel aber kann erstere enthüllen, das für letztere immer ein Rätsel hätte bleiben müssen”.

67 As in vegetation science, especially plant sociology, this problem is sometimes further complicated when it comes to the classification of ecological units. Here we find three levels: the *empirical object*: i.e. the pattern of organisms in a given place, the *entity:* how a particular unit is "carved out" or identified from this pattern ("entitation", Mueller-Dombois and Ellenberg 1974, p. 33f), and the *classification scheme* by which particular objects, understood as an entity of a particular kind (e.g. association, biocoenosis), are assigned to classes using specific criteria. The steps of entitation and classification may sometimes be the same and sometimes not, such as when an empirical object displays only patterns with very specific properties (such as self-regulation), enabling it to be called a unit, *before* it can be classified (see Jax 2002, pp. 110–119).

68 See also the critique by Hutchinson (1967) explaining why he refrained from using the German definition of biocoenoses, as described in FN 6 above.

69 The influence of the different disciplinary backgrounds (zoology vs. botany) in the different countries is an interesting topic, one worth investigating in more detail; clearly, such an exploration lies beyond the scope of this paper.

70 Even the question of what "holistic" means is answered in quite different ways within ecology (Wilson 1988; Bergandi 2011) and in general. As Trepl and Voigt (2011, p. 48) conclude: "It is probably true to say that what links all those things together that are referred to as holism is not much more than the principle that the 'whole' has 'priority' over the 'parts'—whatever 'priority' means exactly—and a set of reservations about any form of 'simplification'".
