# Correction to: “Organismic” positions in early German-speaking ecology and its (almost) forgotten dissidents

**DOI:** 10.1007/s40656-021-00464-w

**Published:** 2021-09-24

**Authors:** Kurt Jax

**Affiliations:** 1 Department of Conservation Biology, Helmholtz-Centre for Environmental Research - UFZ, Permoserstraße 15, 04318 Leipzig, Germany; 2 Department Ecology and Ecosystem Management, Technical University of Munich, Emil-Ramann-Str 6, 85354 Freising, Germany

## Correction to: HPLS (2020) 42:44 10.1007/s40656-020-00328-9

The article *“Organismic” positions in early German-speaking ecology and its (almost) forgotten dissidents*, written by Kurt Jax, was originally published online without Open Access. After publication in volume 42, issue 4, ID 44, it has been decided to make the article an Open Access publication. Therefore, the copyright of the article has been changed to © The Author(s) 2020 and the article is forthwith distributed under the terms of the Creative Commons Attribution.

Open access funding enabled and organized by Projekt DEAL.

The original article has been corrected.

